# The Status and Influencing Factors of Health Behavior Self‐Management in Children With Type 1 Diabetes Mellitus: A Cross‐Sectional Study

**DOI:** 10.1002/hsr2.70700

**Published:** 2025-04-18

**Authors:** Jin‐Xia Yang, Yue Liu, Su‐ying Cao, Guo‐ying Wang, Hui‐min Zhou, Ya‐yun Wang, Zi‐sheng Ai

**Affiliations:** ^1^ Basic Medical Science Tongji University School of Medicine Shanghai China; ^2^ Department of Endocrine Genetic Metabolism Children's Hospital of Soochow University Suzhou China; ^3^ Department of Orthopedics Gongli Hospital Shanghai China

**Keywords:** cross‐sectional study, health behavior, influence factor analysis, nursing, self‐management, Type 1 diabetes mellitus

## Abstract

**Background and Aims:**

To investigate the status of self‐management of health behaviors in children with Type 1 diabetes mellitus and to analyze their influencing factors. Self‐management skills are essential for disease management in children with Type 1 diabetes.

**Methods:**

This cross‐sectional study was conducted on 132 children with Type 1 diabetes mellitus hospitalized in the Department of Endocrinology and Genetic Metabolism of a tertiary children's hospital. Children were selected from September 2023 to March 2024 by convenience sampling method. A general information questionnaire and the Type 1 Diabetes Behavioral Rating Scale were used to conduct the questionnaire survey.

**Results:**

The mean of health behavior self‐management score was (0.60 ± 0.15), and the mean scores of the four dimensions were: daily care behaviors (0.74 ± 0.16), adjustment of diabetes care behaviors (0.29 ± 0.24), diabetes care behavior intervention (0.52 ± 0.25), and other diabetes care behaviors (0.61 ± 0.27) points; one‐way analysis of variance showed that the differences in self‐management scores of health behaviors of children with Type 1 diabetes mellitus were statistically significant when comparing children with different ages, years since diagnosis, whether they were only children, family structure, whether there was a family member with a diagnosis of diabetes mellitus, literacy level of the parents, and average monthly family income (*p* < 0.05); the differences between age, whether they were only children, family structure, whether there was a family member with a diagnosed with diabetes mellitus, and average monthly family income were influential factors in the self‐management of health behaviors of children with Type 1 diabetes mellitus (*p* < 0.05).

**Conclusion:**

The level of health behavior self‐management of children with Type 1 diabetes mellitus is in the middle‐low level, and individualized interventions can be carried out for children with different characteristics to promote the improvement of children's ability to manage their disease and improve their quality of life.

## 1 Introduction

Type 1 diabetes mellitus (T1DM) is a lifelong chronic disease caused by genetic susceptibility, pancreatic β‐cell damage resulting in absolute deficiency of insulin secretion, also known as insulin‐dependent diabetes mellitus [1]. Studies have shown that the disease is prevalent in children and adolescents between the ages of 10 and 14 years [2], and patients under the age of 18 years account for 3/4 of currently diagnosed T1DM patients [3]. the SEARCH study in the United States showed that the average annual incidence of diabetes mellitus among 0–19 years olds between 2002 and 2009 was 25.5/(100,000) [4]; Catanzariti et al. [5] reported that the corrected mean annual incidence of diabetes mellitus in Australian children aged 0–14 years was 21.6/(100,000) in the period 2000–2006; and the average annual incidence of diabetes mellitus among children aged 0–19 years was 25.5/(100,000) in the period 2002–2009 [5]. The latest data from the International Diabetes Federation (IDF) show that 128,900 new cases of children with T1DM occur each year worldwide, and the incidence of pediatric T1DM in China ranks 4th in the world [6]. A study on the incidence and trend of T1DM in China between 2013 and 2018 showed that the annual incidence of T1DM increased from 3.10/(100,000) to 3.60/(100,000) in 6 years [7]. The therapeutic goal of diabetes mellitus is to keep the patient's blood glucose as close to normal as possible and delay the occurrence of complications [8]. The glycemic control of children with T1DM relies on the co‐management of parents and children, and the self‐management of health behaviors is particularly important. Good self‐management can reduce the magnitude of the fluctuation of the patient's blood glucose, reduce the incidence of complications, and improve the patient's quality of survival [9, 10, 11]. Therefore, this study aims to investigate the status of health behavior self‐management and its influencing factors in children with Type 1 diabetes mellitus, with a view to providing a reference for the development of targeted interventions.

## 2 Methods

This cross‐sectional study was conducted in the in a 45‐bed inpatient Endocrinology and Genetic Metabolism ward of a tertiary children's hospital. The Ethics Committee of Children's Hospital of Soochow University approved the study (2024CS141). Written informed consent was obtained from children's parents before entering the study.

### 2.1 Subjects of Investigation

Using convenience sampling method, children with Type 1 diabetes mellitus hospitalized in the Department of Endocrinology and Genetic Metabolism of a tertiary children's hospital and their parents in China were selected from September 2023 to March 2024 as the survey subjects. Inclusion criteria for children: (i) Meet the diagnostic criteria of Chinese T1DM Diagnosis and Treatment Guidelines (2011 edition); (ii) Insulin injection therapy ≥ 4 times a day, disease duration ≥ 6 months; (iii) Age ≤ 18 years old; Exclusion criteria for children: (i) People with speech or communication disorders; (ii) People with psychiatric disorders or other serious physiological diseases. Inclusion criteria for parents: (i) age ≥ 18 years; (ii) caring for the children for more than 7 h per day; (iii) voluntary participation in the study. Exclusion criteria for parents: suffering from mental diseases (Depression, Anxiety Disorders, Schizophrenia and so on), cognitive disorders (Alzheimer's Disease, Dementia, Traumatic Brain Injury and so on), and communication barriers (Language Disorders, Speech Disorders, Hearing Impairments and so on).

### 2.2 Survey Instrument

#### 2.2.1 General Information Questionnaire

It was designed by the researcher, including the child's gender, age, years of diagnosis, whether he/she is an only child, family structure, city, place of residence, whether there is a family member with a diagnosis of diabetes mellitus, mother or father, parent's education level, and the family's average monthly income.

#### 2.2.2 Behavioral Rating Scale for Type 1 Diabetes Mellitus

The Diabetes Behavior Rating Scale (DBRS) developed by McNabb in 1994 was used to evaluate children's diabetes self‐management behavior. The scale was used to assess the frequency with which parents of children with Type 1 diabetes over the age of 8 years implemented 35 diabetes self‐management behaviors at home and the degree to which the child assumed responsibility for implementing these behaviors. The scale has 4 dimensions: Daily Nursing Behavior, Diabetes Care Behavior Modification, Behavioral Interventions for Diabetes Care, Other diabetes care behaviors. There are 2 versions of the scale, insulin pump (37 entries) and insulin pen (36 entries), each with a content‐equivalent adolescent version (filled out by children greater than 8 years of age or older) and a parent version (filled out by parents of children less than or equal to 8 years of age or younger), and the scores for items 4, 5, and 28 in the insulin‐pen‐scored version and for items 4, 5, and 29 in the insulin‐pump‐scored version are calculated using a reverse calculation method (so that a higher score indicate more successful management), and 35 and 36 are marked (did not occur) and may not be included in the calculation of the score. The scale was scored on a Likert scale, with scores for 5 options (scores of 0–4) divided by 4; scores for 6 options (scores of 0–5) divided by 5, and finally the scores for all items were averaged to calculate the mean, with higher scores indicating better self‐management.

### 2.3 Data Collection and Quality Control Methods

(1) Pre‐survey preparation: by the unified training of the researcher using a unified guide to the survey respondents in detail about the purpose of the study; obtaining informed consent from parents, obtaining assent from the child. (2) The survey process: before filling out the researcher explained the questionnaire precautions to the child and parents, filled out by the child and the child's parents in accordance with the actual situation of the child, doubtful by the researcher to make no personal choice tendency to explain, to assist the child or parents to fill out the questionnaire, all questionnaires are filled out on the spot and submitted. (3) Post‐survey data organization: all questionnaire data were exported from the questionnaire star by the researcher and then double‐checked to ensure the accuracy of the data.

This study was conducted in strict accordance with the inclusion and exclusion criteria for the selection of research subjects, by a uniformly trained researcher using a unified instruction language, to the subject of the investigation to introduce in detail the purpose of the study, the significance of the study and parts of the research process that require patient participation, Written informed consent was obtained from children's parents before entering the study to obtain the informed consent of the children's parents, through the collection of sweeps to fill out the questionnaire star questionnaire, before filling out the questionnaire by the researcher to explain to the child and the parents to fill out the questionnaire methods and Before filling out the questionnaire, the researcher explained to the child and parents how to fill out the questionnaire and the precautions to be taken, and the child and parents filled out the questionnaire according to the actual situation of the child, and when in doubt, the researcher explained to the child without any personal choice tendency, and assisted the child or parents to fill out. All questionnaires were completed and submitted on the spot, and all questionnaire data were exported by the researcher and then double‐checked to ensure the accuracy of the data.

### 2.4 Statistical Methods

All data were analyzed using SPSS Statistics for Windows, Version 20.0 (IBM Corp; Armonk, NY). DBRS with scores for 5 options (scores of 0–4) divided by 4; scores for 6 options (scores of 0–5) divided by 5, and finally the scores for all items were averaged to calculate the mean. To assess the normal distribution of continuous variables, the Kolmogorov–Smirnov test was utilized. Descriptive statistics were utilized to check the frequency distribution, mean, and standard errors of the mean. To compare self‐management of health behaviors in children with Type 1 diabetes according to the desired characteristics, independent *t*‐test or one‐way ANOVA was used for within‐ and between‐group mean differences. And non‐parametric tests were used (Mann–Whitney *U*‐test, Kruskal–Wallis *H*‐test). Finally, multifactorial analysis was performed using multiple linear regression analysis. A *p* < 0.05 (two‐sided) was regarded as statistically significant [12].

## 3 Results

### 3.1 General Information of the Survey Respondents

A total of 132 questionnaires were distributed and 132 valid questionnaires were recovered, all questionnaires were included in the analysis. The mean age of the children was (8.64 ± 4.24) years; 36.4% (40 cases) were boys and 63.4% (84 cases) were girls. The general information of 132 children with Type 1 diabetes mellitus is shown in Table 1.

**Table 1:** General information of children with Type 1 diabetes mellitus (*N* = 132).

Variables	Categorization	Statistical value	Variables	Categorization	Statistical value
Sex (number [%])	Female	48 (36.4%)	Residence	City	68 (51.5%)
	Male	84 (63.6%)		Town	28 (21.2%)
Age		8.64 ± 4.24		Rural	36 (27.3%)
Years of diagnosis		1.25 ± 1.67	Family history of diabetes	Yes	28 (21.2%)
Only child	Yes	48 (36.4%)		No	104 (78.8%)
	No	84 (63.6%)	Primary caregiver	Parents	120 (90.9%)
Family structure	Three generations	104 (78.8%)		Grandparent	12 (9.1%)
	Two parents	24 (18.2%)		Other	0
	Single parent	4 (3.0%)	Educational level of primary caregiver	Junior high school and below	24 (18.2%)
Ethnicity	Han Chinese	132 (100%)		High school/junior college	44 (33.3%)
	Other	0		College	40 (30.3%)
City	Suzhou	104 (78.8%)		Undergraduate	20 (15.2%)
	Wuxi	12 (9.1%)		Graduate student and above	4 (3.0%)
	Taizhou	4 (3.0%)	Average monthly family income	Below 3000 RMB	16 (12.1%)
	Lianyungang	4 (3.0%)		3000–6000 RMB	36 (27.3%)
	Other	8 (6.1%)		6000–10,000 RMB	48 (36.4%)
				Above 10,000 RMB	32 (24.2%)

### 3.2 Score of Self‐Management of Health Behaviors in Children With Type 1 Diabetes Mellitus

The health behavior self‐management score of 132 children with Type 1 diabetes mellitus was (0.60 ± 0.15), and the scores of the four parts of the scale were: Daily Nursing Behavior (0.74 ± 0.16), Diabetes Care Behavior Modification (0.29 ± 0.24), Behavioral Interventions for Diabetes Care (0.52 ± 0.25), and Other diabetes care behaviors (0.61 ± 0.27) scores, as shown in Table 2.

**Table 2:** Scores of self‐management of health behaviors in children with Type 1 diabetes (*N* = 132).

Variables		$\bar{x}$ ± s	
Daily nursing behavior			
1. create a meal plan based on your child's own situation?		0.64 ± 0.31	
2. weigh or measure food?		0.70 ± 0.32	
3. use food labels to organize meals?		0.65 ± 0.34	
4. fat intake above the upper limit recommended by the diet plan or doctor?		0.58 ± 0.35	
5. consume more sugar than the diet plan or the upper limit recommended by your doctor?		0.69 ± 0.30	
6. inject insulin correctly at the dose prescribed by your doctor (including dose adjustments based on diet and blood glucose levels)?		0.84 ± 0.21	
7. inject insulin at the prescribed time?		0.87 ± 0.16	
8. record insulin injections in a logbook?		0.80 ± 0.29	
9. use the correct type of insulin injection?		0.90 ± 0.15	
10. perform insulin injection site rotation?		0.87 ± 0.14	
11. measure blood glucose at the frequency recommended by your doctor?		0.77 ± 0.32	
12. measure blood glucose at the prescribed time?		0.80 ± 0.28	
13. record your child's blood glucose values in a log or chart?		0.78 ± 0.31	
14. your child carries “fast‐absorbing sugars” (e.g., candy, juice) with him/her?		0.66 ± 0.34	
15. your child exercises or participates in sports for more than 20 min a day?		0.77 ± 0.27	
16. your child carries an item that identifies him/her as having diabetes (e.g., first aid card)?		0.37 ± 0.39	
Diabetes care behavior modification			
17. monitor blood glucose at each meal (including main meals, fruit, snacks, etc.)?		0.80 ± 0.29	
18. the number of times the amount of food and drink is adjusted accordingly when the amount of exercise is changed?		0.25 ± 0.29	
19. the number of times the insulin dose was adjusted accordingly when the amount of exercise was changed?		0.24 ± 0.31	
20. the number of times the insulin dose was adjusted accordingly when eating more or less than usual?		0.33 ± 0.35	
21. How many times was the amount of exercise changed when blood glucose was higher or lower than usual?		0.33 ± 0.33	
22. the number of times the insulin dose was adjusted accordingly when blood glucose did not reach the target range?		0.38 ± 0.34	
23. How many times has your child received help for diabetes at school, home, or in social situations?		0.18 ± 0.28	
Behavioral interventions for diabetes care			
24. measuring blood glucose in a timely manner?		0.54 ± 0.37	
25. intake of “fast‐absorbing sugars” such as candy within 10 min?		0.37 ± 0.37	
26. measuring blood glucose again within 20 min of consuming fast‐absorbing candy?		0.39 ± 0.37	
27. eat a normal diet after consuming the required amount of fast‐absorbing sugar?		0.40 ± 0.37	
28. over‐eat as a result of hypoglycemia?		0.75 ± 0.34	
29. measure blood glucose in a timely manner?		0.62 ± 0.35	
30. adjust insulin dosage based on blood glucose measurements?		0.59 ± 0.38	
Other diabetes care behaviors			
31. correctly adjust insulin dose when eating out (e.g., at restaurants, parties)?		0.58 ± 0.36	
32. inform your child's friends, teachers, coaches, and others how to handle low blood sugar situations?		0.57 ± 0.36	
33. tell the school nurse, dentist and eye doctor that your child has diabetes?		0.67 ± 0.35	
34. visit the clinic or doctor regularly for follow‐up visits?		0.70 ± 0.30	
35. seek help from a health care provider to adjust insulin dosage due to frequent high or low blood sugar?		0.56 ± 0.40	
36. contact a health care provider when your child has severe symptoms of diabetes (e.g., drinking too much, needing large amounts of “fast‐absorbing sugar”)?		0.57 ± 0.40	
overall assessment	0.60 ± 0.15	

### 3.3 One‐Way Analysis of Variance of Health Behavior Self‐Management Scores Among Children With Type 1 Diabetes Mellitus

One‐way analysis of variance analysis showed that there was a statistical difference in health behavior self‐management scores of children with Type 1 diabetes mellitus between different ages (*F* = 3.759, *p* = 0.0.026), years of diagnosis (*F* = 16.972, *p* < 0.0.001), whether they were only children (*F* = 5.145, *p* = 0.0.025), family structure (*F* = 10.991, *p* < 0.0.001), whether there was a family member with a diagnosis of diabetes mellitus (*F* = 12.825, *p* < 0.0.001), educational level of the parents (*F* = 3.209, *p* = 0.015), and average monthly family income (*F* = 9.194, *p* < 0.001), as shown in Table 3.

**Table 3:** Univariate analysis of health behavior self‐management scores of children with Type 1 diabetes (*N* = 132).

Variables	Categorization	Score	*F*	*p* value
Sex	Female	0.59 ± 0.14	0.507	0.478
	Male	0.60 ± 0.16		
Age (years)	< 3	0.51 ± 0.06	3.759	0.026*
	[3–7]	0.58 ± 0.15		
	7>	0.63 ± 0.15		
Years of diagnosis	< 1	0.64 ± 0.12	16.972	< 0.001**
	[1–3]	0.50 ± 0.13		
	3>	0.67 ± 0.18		
Only child	Yes	0.55 ± 0.11	5.145	0.025*
	No	0.63 ± 0.17		
Family structure	Three generations	0.57 ± 0.13	10.991	< 0.001**
	Two parents	0.72 ± 0.20		
	Single parent	0.53 ± 0.00		
Ethnicity city	Han Chinese	0.60 ± 0.16	0.479	0.751
	Other	0.58 ± 0.19		
	Suzhou	0.66 ± 0.12		
	Wuxi	0.53 ± 0.11		
	Taizhou	0.57 ± 0.06		
Residence	City	0.60 ± 0.15	2.456	0.090
	Town	0.65 ± 0.21		
	Rural	0.56 ± 0.76		
Family history of diabetes	Yes	0.61 ± 0.21	12.825	< 0.001**
	No	0.59 ± 0.13		
parents	Parents	0.61 ± 0.16	2.717	0.102
	Grandparent	0.53 ± 0.02		
	Other	0.60 ± 0.15		
Educational level of parents	Junior high school and below	0.57 ± 0.09	3.209	0.015*
	High school/junior college	0.62 ± 0.15		
	College	0.55 ± 0.14		
	Undergraduate	0.66 ± 0.22		
	Graduate student and above	0.73 ± 0.10		
Average monthly family income	Below 3000 RMB	0.49 ± 0.13	9.194	< 0.001**
	3000–6000 RMB	0.61 ± 0.12		
	6000–10,000 RMB	0.56 ± 0.11		
	Above 10,000 RMB	0.70 ± 0.19		

*Note:* * is *p* value < 0.05, ** is *p* value < 0.01.

### 3.4 Multifactorial Analysis of Health Behavior Self‐Management Scores of Children With Type 1 Diabetes Mellitus

Taking the dependent variable of health behavior self‐management score of children with Type 1 diabetes mellitus and the seven characteristic variables that were statistically significant in the univariate analysis as independent variables, multivariate linear regression analysis was used to explore the influencing factors of health behavior self‐management of children with Type 1 diabetes mellitus (*α*_in_ = 0.05, *α*_out_ = 0.10), and the way of assigning the independent variables is shown in Table 4. The results showed that age, whether they were only child, family structure, whether family members have diagnosed diabetes or not, and average monthly family income were the influencing factors of self‐management of health behaviors of children with Type 1 diabetes (*p* < 0.05), as shown in Table 5. It was suggested that the older the age, the longer the number of years since diagnosis, the more educated the parents, and the higher the monthly family income, the higher the children's health behavior self‐management scores were; and the health behavior self‐management scores were higher in children who were not only children, in two‐parent families, and in children who had a family member with a diagnosis of diabetes mellitus, compared to the other groups.

**Table 4:** Table of variable assignments.

Self‐variable	Assignment method
Age (years)	< 3 = 1, [3–7] = 2, 7 > = 3
Years of diagnosis	< 1 = 1, [1–3] = 2, 3 > = 3
Only child	Yes = 1, No = 2
Family structure	Three generations = 1, Two parents = 2, Single parent = 3
Family history of diabetes	Yes = 1, No = 2
Educational level of parents	Junior high school and below = 1, High school/junior college = 2, College = 3, Undergraduate = 4, Graduate student and above = 5
Average monthly family income	Below 3000 RMB = 1, 3000–6000 RMB = 2, 6000–10,000 RMB = 3, Above 10,000 RMB = 4

**Table 5:** Results of multiple stepwise regression analysis of self‐management health behavior scores of children with Type 1 diabetes mellitus.

Variables	Regression coefficient	Standard error	Standard regression coefficient	*t*	*p* value
A constant (math.)	−0.164	0.118		−1.387	0.168
Age	0.073	0.019	0.289	3.79	< 0.001
Only child	0.103	0.024	0.326	4.347	< 0.001
Family structure	0.081	0.024	0.263	3.336	0.001
Family history of diabetes	0.075	0.031	0.203	2.459	0.015
Average monthly family income	0.063	0.014	0.403	4.432	< 0.001

*Note: R* ^2^ = 34.2%, adjusted *R* ^2^ = 30.5%, *F* = 9.213, *p* value < 0.001.

## 4 Discussion

This study found that children with Type 1 diabetes mellitus have a moderate level of self‐management of health behaviors, and age, whether they are only child, family structure, whether there is a family member with diagnosed diabetes mellitus, and average monthly family income are the factors influencing the self‐management of health behaviors of children with Type 1 diabetes mellitus. The results of this study suggest that individualized interventions should be implemented for children with Type 1 diabetes with different individual characteristics to promote the children's disease management ability and quality of life.

### 4.1 Self‐Management of Health Behaviors in Children With Type 1 Diabetes Needs Further Improvement

With the consequent changes in lifestyle and dietary structure, the age of onset of diabetes mellitus has shown a younger development trend. The treatment of diabetes mellitus includes dietary control, medication and metabolic monitoring, etc., which cannot be separated from the patient's self‐management behavior, so self‐management is an important factor affecting the treatment effect and survival quality of children with T1DM. The findings of this study showed that 132 children with Type 1 diabetes mellitus had a health behavior self‐management score of (0.60 ± 0.15), which was close to the median of 0.5. Compared with previous studies, the highest mean score for all dimensions of self‐management skills in children with diabetes was (3.27 ± 0.49), which is closer to the full 4 points, it shows that there is room for this score to increase, indicating that their health behavior self‐management ability needs to be further improved [13]. The reason for this is mainly because children with diabetes are younger than adults, have poor cognition, low treatment compliance, lack a deep understanding of their own disease, and some of them are unable to accurately express their feelings, care needs, hypoglycemia and other adverse symptoms in a timely manner, which is very likely to lead to significant fluctuations in blood glucose. Domestic scholars He Yangyang et al [13] used the adolescent Type 1 diabetes mellitus self‐management scale to investigate and found that children with diabetes mellitus self‐management scale in the daily care of the highest score, diabetes‐related problem solving scored the lowest; another survey showed that the T1DM children's self‐management of health behaviors is unsatisfactory [14, 15], which is also in line with the results of this study. Based on this, for children with Type 1 diabetes mellitus, researchers can develop measures to improve the self‐management of children's health behaviors and provide targeted interventions from the persistent diabetes education and training, the development of individualized treatment plans, self‐monitoring and self‐regulation, psychological support, and regular follow‐up and assessment.

### 4.2 Children With Type 1 Diabetes Mellitus Are Affected by a Variety of Individual Factors

#### 4.2.1 The Older the Child, the Higher the Level of Self‐Management of Health Behaviors

The results of this study show that the older the child, the higher the level of self‐management of health behaviors (*F* = 3.759, *p* < 0.05), and the multiple regression coefficient is 0.073. This is consistent with the results of the international research, Pierce et al. [16] showed that subjective and objective support, refusal parenting, warm parenting is an influential factor in the level of self‐management of the child. The reason for this is that, on the one hand, with age, children's maturity increases, independence increases, and cognitive ability and self‐control increases so that they are more able to understand the importance of the disease and the necessity of self‐management, and children also gain more autonomy and self‐decision‐making ability, and are able to carry out behaviors such as blood glucose monitoring, insulin injection, and controlling their diets more autonomously; on the other hand, with age, children's experience continues to accumulate, and children's experience continues to grow. On the other hand, with the growth of age, children's experience accumulates, social support increases, and the experience gathered during the period of illness and treatment makes children more familiar with the characteristics of the disease, treatment methods and self‐management skills, and at the same time, they can get more support from their families, schools and health care teams, which can promote the improvement of their self‐management ability.

#### 4.2.2 The Level of Health Behavior Self‐Management of Non‐Only‐Children Is Higher Than That of Only‐Children

The results of this study showed that the level of self‐management of health behaviors of non‐only children was higher than that of only children (*F* = 5.145, *p* < 0.05), with a multiple regression coefficient of 0.103. This is consistent with the results of the international research, de Cássia [17] showed that non‐only children may have more siblings in the family, which provides them with additional social support and opportunities for emotional interaction. The reasons for this are that, on the one hand, thanks to the support and assistance of the family, children who are not only children are usually able to receive additional support and assistance from their siblings, and the care and supervision of the family members might be helpful to ensure that children adhere to the health behaviors and treatment plans, and that children in non‐only families might be inspired by siblings and help to form a health behavior and treatment plan. health behaviors and treatment plans, and in non‐only‐child families, children may be inspired by their siblings to learn from and motivate each other, which may help to develop a joint effort in self‐management of health behaviors; on the other hand, due to the dispersed attention of the family, non‐only‐child children with diabetes may not have received overly focused attention in the family, and therefore need to be independent and self‐managed more quickly to improve self‐management skills.

#### 4.2.3 The Level of Health Behavior Self‐Management of Those With Two‐Parent Family Structure Is Higher Than Those With Other Family Structure

The results of this study show that the level of self‐management of health behaviors of two‐parent families is higher than that of other families (*F* = 10.991, *p* < 0.001), and the multiple regression coefficient is 0.081. Pre‐adolescent and adolescent children are in a transition period from early childhood to adulthood, and they are in a critical period when they must cope with a lot of problems in their families, friends, schools, and health. Family environment plays a decisive role in the formation of personality and coping styles of children and adolescents. The family environment plays a decisive role in the formation of personality and coping styles of children and adolescents, and studies have confirmed that self‐management of children with T1DM is closely related to family functioning and social support [18, 19, 20, 21, 22]. Parents are primarily responsible for their children's disease management, and self‐management in children and adolescents with Type 1 diabetes mellitus has been defined as an active, daily, and flexible process in which children share responsibility and make decisions with their parents to better manage their disease and achieve health and well‐being, reflecting a developmental trajectory from parental dependence to parental cooperation. The Chinese Guidelines for the Diagnosis and Management of Type 1 Diabetes Mellitus [23] recommend that once T1DM is diagnosed, health education should be provided to the patient and at least one family member to guide them in the self‐management of the disease. In fact, parents in two‐parent family structure pay more attention to the management of children's diseases and provide more emotional support, which may also be one of the reasons for the better self‐management ability of health behaviors in two‐parent family structure.

#### 4.2.4 Self‐Management of Health Behaviors Was Higher Among Family Members With a Diagnosis of Diabetes Mellitus Than Those Without a Diagnosis

The results of this study showed that the level of health behavior self‐management of family members with diagnosed diabetes mellitus was higher than that of those without diagnosed diabetes mellitus (*F* = 12.825, *p* < 0.001), with a multiple regression coefficient of 0.075. The McCollum study findings are consistent with this [24]. The reason for this may be, on the one hand, due to the awareness of the family and the education on the disease, the fact that there are already diabetes mellitus patients in the family usually means that the family has a higher level of awareness and knowledge of diabetes mellitus and is able to provide more comprehensive disease education to the children. On the other hand, it may be due to the role model effect and the sharing of common management experience. The sharing of experience of existing diabetic patients in the family will form a common mode of disease management for the whole family, from which the children can benefit and better self‐manage.

#### 4.2.5 The Higher the Average Monthly Household Income, the Higher the Level of Health Behavior Self‐Management

The results of this study show that the higher the average monthly family income, the higher the level of self‐management of health behaviors (*F* = 9.194, *p* < 0.001), with a multiple regression coefficient of 0.063. The Herbert study findings are consistent with this [25]. The reason for this is that, on the one hand, thanks to the access to health care resources and information, higher family income usually means better access to health care resources and information, and the family can obtain quality health care and information on disease management more easily, which encourages the children's self‐management. On the other hand, due to the reduction of economic and psychological pressure, higher family income may mean better living conditions and family environment, which may help to reduce the psychological pressure of the children, and thus be more conducive to positively facing the disease and adopting positive self‐management behaviors.

### 4.3 Individualized Interventions for Children With Type 1 Diabetes Mellitus With Different Individual Characteristics Are Recommended

Ninety‐eight percent of children with diabetes mellitus are T1DM, which requires lifelong therapeutic management with glucose monitoring, insulin injection, diet, exercise and health education [26]. If blood glucose is not effectively controlled, it often leads to acute and chronic complications such as hypoglycemic coma and ketoacidosis, which can even be life‐threatening in severe cases [27]. The results of this study showed that age, whether the child is an only child, family structure, whether there is a family member with diagnosed diabetes, and average monthly family income are the factors influencing the self‐management of health behaviors of children with Type 1 diabetes (*p* < 0.05). This also suggests that the researcher should consider various factors such as children's physical, psychological, and social environments to individualize the intervention for children with Type 1 diabetes with different individual characteristics. For example, considering the age and developmental stage of the children, for young children, the intervention may need to focus on family education and guardian involvement, with emphasis on dietary control and insulin injection techniques. For adolescents, interventions may need to incorporate psychosocial support, emphasizing communication and support with peers and encouraging active participation in self‐management, as well as providing information about managing changing physical as well as emotional aspects; or, for example, considering the family support system, interventions for children with varying levels of family support may include providing family support, educating family members about how they can help the child to manage his or her diabetes, and providing resources to help them understand and cope with their child's disease. In summary, individualized interventions require a comprehensive understanding of many aspects of the child's characteristics and close communication with the child and his or her family to develop an effective individualized intervention plan.

## 5 Limitations

This study was conducted at only one research site with a limited sample size, and convenience sampling method may cause selection bias. In the future, we plan to conduct a multi‐center, large‐sample study with a view to comparing the self‐management ability of children in different regions and years of diagnosis, so that individualized intervention programs can be targeted to help children and families better manage their disease and promote recovery.

## 6 Conclusion

This study found that children with Type 1 diabetes mellitus have a moderate level of self‐management of health behaviors, and that age, whether they are only child, family structure, whether there is a family member with diagnosed diabetes mellitus, and average monthly family income are the factors influencing self‐management of health behaviors of children with Type 1 diabetes mellitus; the results of the study suggest that individualized interventions should be carried out for children with Type 1 diabetes mellitus with different individual characteristics to promote the ability of disease management and quality of life of the children. The results suggest that individualized interventions should be implemented for children with Type 1 diabetes with different individual characteristics to promote the children's disease management ability and quality of life.

## Author Contributions

**Jin‐Xia Yang:** funding acquisition, resources, software, supervision, validation, visualization, writing – original draft. **Yue Liu:** resources, software, supervision, validation, visualization, writing – review and editing. **Su‐ying Cao:** conceptualization, data curation, formal analysis, investigation, methodology. **Guo‐ying Wang:** conceptualization, data curation, formal analysis, resources, visualization. **Hui‐min Zhou:** methodology, project administration, validation, visualization. **Ya‐yun Wang:** investigation, methodology, validation, visualization. **Zi‐sheng Ai:** formal analysis, funding acquisition, investigation, software, supervision, validation.

## Conflicts of Interest

The authors declare no conflicts of interest.

## Transparency Statement

The lead author Su‐ying Cao, Zi‐sheng Ai affirms that this manuscript is an honest, accurate, and transparent account of the study being reported; that no important aspects of the study have been omitted; and that any discrepancies from the study as planned (and, if relevant, registered) have been explained.

## Data Availability

The authors confirm that the data supporting the findings of this study are available within the article.
